# Effectiveness of COVID-19 Vaccines in Preventing Hospitalization Among Adults Aged ≥65 Years — COVID-NET, 13 States, February–April 2021

**DOI:** 10.15585/mmwr.mm7032e3

**Published:** 2021-08-13

**Authors:** Heidi L. Moline, Michael Whitaker, Li Deng, Julia C. Rhodes, Jennifer Milucky, Huong Pham, Kadam Patel, Onika Anglin, Arthur Reingold, Shua J. Chai, Nisha B. Alden, Breanna Kawasaki, James Meek, Kimberly Yousey-Hindes, Evan J. Anderson, Monica M. Farley, Patricia A. Ryan, Sue Kim, Val Tellez Nunez, Kathryn Como-Sabetti, Ruth Lynfield, Daniel M. Sosin, Chelsea McMullen, Alison Muse, Grant Barney, Nancy M. Bennett, Sophrena Bushey, Jessica Shiltz, Melissa Sutton, Nasreen Abdullah, H. Keipp Talbot, William Schaffner, Ryan Chatelain, Jake Ortega, Bhavini Patel Murthy, Elizabeth Zell, Stephanie J. Schrag, Christopher Taylor, Nong Shang, Jennifer R. Verani, Fiona P. Havers

**Affiliations:** ^1^CDC COVID-19 Response Team; ^2^Epidemic Intelligence Service, CDC; ^3^General Dynamics Information Technology, Falls Church, Virginia; ^4^California Emerging Infections Program, Oakland, California; ^5^School of Public Health, University of California, Berkley, California; ^6^Colorado Department of Public Health & Environment; ^7^Connecticut Emerging Infections Program, Yale School of Public Health, New Haven, Connecticut; ^8^Emory University School of Medicine, Atlanta, Georgia; ^9^Georgia Emerging Infections Program, Georgia Department of Public Health; ^10^Atlanta Veterans Affairs Medical Center, Atlanta, Georgia; ^11^Maryland Department of Health; ^12^Michigan Department of Health & Human Services; ^13^Minnesota Department of Health; ^14^New Mexico Emerging Infections Program, New Mexico Department of Health; ^15^New York State Department of Health; ^16^University of Rochester School of Medicine and Dentistry, Rochester, New York; ^17^Ohio Department of Health; ^18^Public Health Division, Oregon Health Authority; ^19^Vanderbilt University Medical Center, Nashville, Tennessee; ^20^Salt Lake County Health Department, Salt Lake City, Utah; ^21^Stat-Epi Associates, Inc., Ponte Vedra Beach, Florida.

Clinical trials of COVID-19 vaccines currently authorized for emergency use in the United States (Pfizer-BioNTech, Moderna, and Janssen [Johnson & Johnson]) indicate that these vaccines have high efficacy against symptomatic disease, including moderate to severe illness (*1*–*3*). In addition to clinical trials, real-world assessments of COVID-19 vaccine effectiveness are critical in guiding vaccine policy and building vaccine confidence, particularly among populations at higher risk for more severe illness from COVID-19, including older adults. To determine the real-world effectiveness of the three currently authorized COVID-19 vaccines among persons aged ≥65 years during February 1–April 30, 2021, data on 7,280 patients from the COVID-19–Associated Hospitalization Surveillance Network (COVID-NET) were analyzed with vaccination coverage data from state immunization information systems (IISs) for the COVID-NET catchment area (approximately 4.8 million persons). Among adults aged 65–74 years, effectiveness of full vaccination in preventing COVID-19–associated hospitalization was 96% (95% confidence interval [CI] = 94%–98%) for Pfizer-BioNTech, 96% (95% CI = 95%–98%) for Moderna, and 84% (95% CI = 64%–93%) for Janssen vaccine products. Effectiveness of full vaccination in preventing COVID-19–associated hospitalization among adults aged ≥75 years was 91% (95% CI = 87%–94%) for Pfizer-BioNTech, 96% (95% CI = 93%–98%) for Moderna, and 85% (95% CI = 72%–92%) for Janssen vaccine products. COVID-19 vaccines currently authorized in the United States are highly effective in preventing COVID-19–associated hospitalizations in older adults. In light of real-world data demonstrating high effectiveness of COVID-19 vaccines among older adults, efforts to increase vaccination coverage in this age group are critical to reducing the risk for COVID-19–related hospitalization.

COVID-NET includes data on laboratory-confirmed COVID-19–associated hospitalizations in 99 U.S. counties in 14 states, representing approximately 10% of the U.S. population.^†^ COVID-NET cases were hospitalizations that occurred in residents of a designated COVID-NET catchment area who were admitted within 14 days of a positive SARS-CoV-2 test result. COVID-NET program personnel collected information on COVID-19 vaccination status (vaccine product received, number of doses, and administration dates) from state IISs for all sampled COVID-NET cases.^§^ Some sites expanded collection of information on vaccination status to all reported COVID-NET cases, not only sampled cases, which were included for analysis if all cases in a single month had vaccination status available. Data from 13 sites were included for analysis; one site (Iowa) does not have access to the state IIS and cannot collect vaccination data.^¶^ Population-level vaccination coverage was determined using deidentified person-level COVID-19 vaccination data reported to CDC by jurisdictions, pharmacies, and federal entities through the IISs,** Vaccine Administration Management System,^††^ or direct data submission.^§§^

The study was restricted to adults aged ≥65 years and included the period February 1–April 30, 2021. The Janssen vaccine was authorized for use during the study period beginning March 15, 2021.^¶¶^ Patients were classified as 1) unvaccinated (no IIS record of vaccination), 2) partially vaccinated (1 dose of Moderna or Pfizer-BioNTech received ≥14 days before hospitalization or 2 doses, with the second dose received <14 days before hospitalization), or 3) fully vaccinated (receipt of both doses of Moderna or Pfizer-BioNTech with second dose received ≥14 days before hospitalization or receipt of a single dose of Janssen ≥14 days before hospitalization). Patients with only 1 dose of any COVID-19 vaccine received <14 days before hospitalization were excluded. Daily county-level coverage data for adults aged 65–74 and ≥75 years in the COVID-NET catchment area were estimated using population denominators from the U.S. Census Bureau; vaccination status was classified as described for hospitalized cases.*** For vaccine records missing county of residence, county of vaccine administration was used.

To estimate vaccine effectiveness and corresponding 95% CIs, methods were adapted based on previously published literature (*4*). Poisson regression was used to compare case counts by vaccination status (outcome) and the proportion of the population vaccinated and unvaccinated (offset).^†††^ Data were stratified by age group because of the potential for confounding by age, and adjusted for COVID-NET site, time (number of weeks since the start of the study period as a categorical covariate), and monthly site-specific sampling frequency.^§§§^ Vaccine effectiveness was calculated as one minus the exponent of the estimated coefficient of the exposure (vaccination status) variable. For estimating effectiveness of full vaccination, partially vaccinated persons were excluded; for estimating effectiveness of partial vaccination, fully vaccinated persons were excluded. Vaccine product–specific estimates excluded persons who had received other COVID-19 vaccines. To account for the interval between infection and hospitalization, sensitivity analyses were conducted using a reference date 1 week and 2 weeks before admission, rather than admission date, for classification of vaccination status for cases (i.e., adding 7 and 14 days, respectively between last vaccine dose and hospital admission date); the same adjustment was included for population vaccination coverage. Statistical analyses were conducted using SAS software (version 9.4; SAS Institute). This activity was reviewed by CDC and was conducted consistent with applicable federal law and CDC policy.^¶¶¶^

During February 1–April 30, 2021, among 7,280 eligible COVID-NET patients, 5,451 (75%) were unvaccinated, 867 (12%) were partially vaccinated, and 394 (5%) were fully vaccinated; 568 (8%) who received a single vaccine dose <14 days before hospitalization were excluded from the analysis (Table). Vaccination coverage in the population increased rapidly during this period among persons aged ≥65 years and varied by age and vaccine product (Figure 1). Among adults aged ≥65 years in the COVID-NET catchment area, full vaccination coverage from any of the three authorized vaccines ranged from 0.7% on February 1, 2021, to 72% on April 30, 2021.

**TABLE:** Hospitalized COVID-19 patients aged ≥65 years, by vaccination status and age group (N = 6,712)* — COVID-NET,**^†^** 13 states, February 1 –April 30, 2021

Vaccination status^§,¶^	No. of cases, by age group (yrs)		
	65–74	≥75	Total (≥65)
All patients (any vaccination status)	3,306	3,406	**6,712**
Unvaccinated patients	2,869	2,582	**5,451**
**Vaccinated patients, by vaccine product**			
**Pfizer-BioNTech**			
Partially vaccinated	188	379	**567**
Fully vaccinated	73	185	**258**
**Moderna**			
Partially vaccinated	104	196	**300**
Fully vaccinated	56	56	**112**
Janssen (Johnson & Johnson)**			
Fully vaccinated	16	8	**24**

**Abbreviation:** COVID-NET = Coronavirus Disease 2019–Associated Hospitalization Surveillance Network.

* Among 7,280 eligible COVID-NET patients, 568 patients (251 aged 65–74 years and 317 aged ≥75 years) who received only 1 dose of any COVID-19 vaccine <14 days before hospitalization were excluded from analysis.

^†^ COVID-NET data included in this analysis were from the following states: California, Colorado, Connecticut, Georgia, Maryland, Michigan, Minnesota, New Mexico, New York, Ohio, Oregon, Tennessee, and Utah.

^§^ Partially vaccinated patients received 1 dose of Moderna or Pfizer-BioNTech vaccine ≥14 days before hospitalization or 2 doses, with the second dose received <14 days before hospitalization.

^¶^ Fully vaccinated patients received both doses of Moderna or Pfizer-BioNTech vaccine, with second dose received ≥14 days before hospitalization, or receipt of a single dose of Janssen (Johnson & Johnson) vaccine ≥14 days before hospitalization.

** The Janssen vaccine was authorized for use after the study began; cases were included during March 15–April 30, 2021.

**FIGURE 1:**
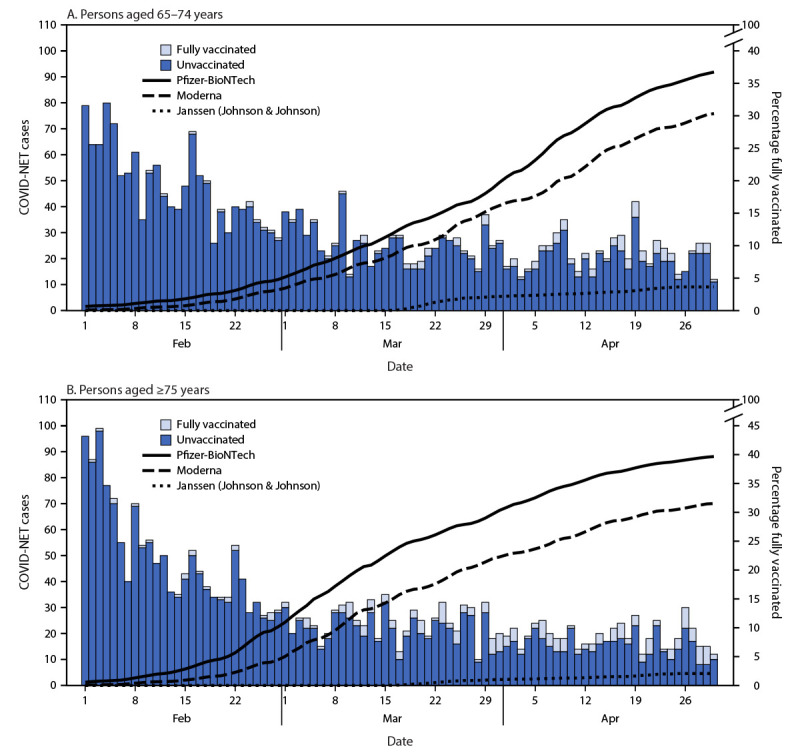
COVID-NET* cases and full vaccination coverage among persons aged 65–74 years (A) and persons aged ≥75 years (B) — 13 states, February 1–April 30, 2021 **Abbreviation:** COVID-NET = Coronavirus Disease 2019–Associated Hospitalization Surveillance Network. * COVID-NET data included in this analysis were from the following states: California, Colorado, Connecticut, Georgia, Maryland, Michigan, Minnesota, New Mexico, New York, Ohio, Oregon, Tennessee, and Utah.

Effectiveness of full vaccination in preventing hospitalization among adults aged 65–74 years was estimated at 96% (95% CI = 94%–98%) for Pfizer-BioNTech, 96% (95% CI = 95%–98%) for Moderna, and 84% (95% CI = 64%–93%) for Janssen vaccine products. Among adults aged ≥75 years, effectiveness of full vaccination was 91% (95% CI = 87%–94%) for Pfizer-BioNTech, 96% (95% CI = 93%–98%) for Moderna, and 85% (95% CI = 72%–92%) for Janssen vaccine products (Figure 2). Effectiveness of partial vaccination among adults aged 65–74 years was 84% (95% CI = 76%–89%) for Pfizer-BioNTech and 91% (95% CI = 87%–93%) for Moderna vaccine products. Among those aged ≥75 years, effectiveness of partial vaccination was 66% (95% CI = 48%–77%) for Pfizer-BioNTech and 82% (95% CI = 76%–86%) for Moderna vaccine products. Sensitivity analyses accounting for interval between infection and hospitalization did not yield notably different vaccine effectiveness estimates, with point estimates varying by <1% for Pfizer-BioNTech and Moderna vaccine models. Point estimates for Janssen COVID-19 vaccine models varied by <10%, with few cases eligible for inclusion and wide CIs.

**FIGURE 2:**
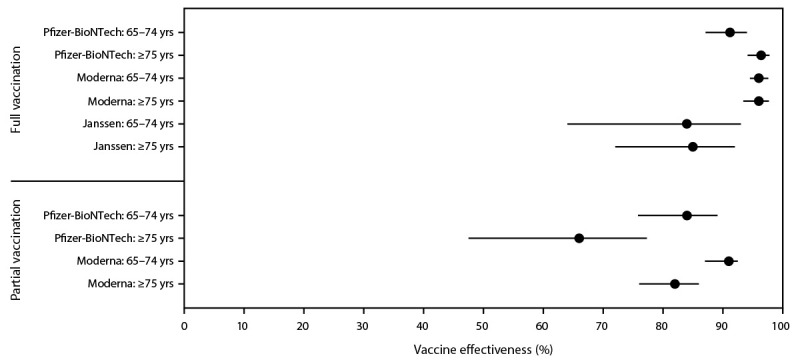
Estimates of vaccine effectiveness in preventing COVID-19–associated hospitalization among patients aged ≥65 years for the COVID-NET catchment area, by vaccine product and age group using the screening method — COVID-NET, 13 states,* February 1–April 30, 2021^†^ **Abbreviations:** COVID-NET = Coronavirus Disease 2019–Associated Hospitalization Surveillance Network; Janssen = Janssen (Johnson & Johnson). * COVID-NET data included in this analysis were from the following states: California, Colorado, Connecticut, Georgia, Maryland, Michigan, Minnesota, New Mexico, New York, Ohio, Oregon, Tennessee, and Utah. ^†^ Confidence intervals indicated by error bars.

## Discussion

In this analysis of 7,280 laboratory-confirmed COVID-19–associated cases among hospitalized adults aged ≥65 years, all three COVID-19 vaccine products currently authorized for use in the United States had high effectiveness in preventing laboratory-confirmed COVID-19–associated hospitalizations. The effectiveness of full vaccination with mRNA vaccines (Pfizer BioNTech and Moderna) was ≥91% and of Janssen was ≥84% among adults aged ≥65 years. These findings are consistent with estimates from other observational studies of the mRNA vaccines and provide an early estimate of the effectiveness of Janssen in preventing COVID-19–associated hospitalization (*1*–*3*,*5*). Although the method used in this analysis does not account for many important potential confounders and results should be interpreted with caution, taken together, these findings provide additional evidence that available vaccines are highly effective in preventing COVID-19–associated hospitalizations and demonstrate that performance of COVID-19 vaccines can be assessed using existing disease surveillance and immunization data.

This analysis provides an early estimate of the Janssen vaccine effectiveness in preventing hospitalization in older adults, adding to the limited observational data available assessing Janssen vaccine effectiveness.**** These findings are consistent with clinical trial efficacy data, which found an efficacy of 76.7% for prevention of moderate to severe disease ≥14 days after vaccination (*3*). The relatively few cases and low population vaccination coverage with Janssen in this analysis likely contributed to the wide CIs for the vaccine effectiveness estimate. In addition, given vaccine prioritization for populations at high risk, older adults receiving the Janssen product were more likely to be at lower risk and differ substantially from those receiving products available earlier in the vaccine rollout. Other observational studies have demonstrated variability in the effectiveness of partial vaccination with mRNA vaccines in preventing hospitalization, with point estimates of effectiveness of 64% to 91% (*5*,*6*). Variation in estimates of effectiveness of partial vaccination between Pfizer-BioNTech and Moderna in this analysis might represent confounding from differences among the persons receiving these products. Residents of long-term care facilities (LTCFs) were prioritized early in the vaccine rollout and were more likely to receive Pfizer-BioNTech than Moderna.^††††^ The underlying risk for severe illness from COVID-19 in this medically fragile population could contribute to lower vaccine effectiveness among LTCF residents than among the general population of older adults and to an apparently lower effectiveness of Pfizer-BioNTech. Moreover, if partial protection increases between the third and fourth week after receipt of the first dose, it is possible that the timing of the second Pfizer-BioNTech and Moderna doses (21 and 28 days after the first dose, respectively) could affect the observed effectiveness of partial vaccination. Therefore, these results should not be interpreted as definitive evidence of a difference in the effectiveness of partial vaccination between the two mRNA vaccines, but rather as an indication that further evaluation is warranted.

The findings in this report are subject to at least four limitations. First, although adjustments were made for time and site, the analysis did not adjust for other potential confounders, such as chronic conditions, because person-level data were not available for the catchment population. In addition, although the analysis was stratified by age and adjusted for time and site, the heterogeneity of disease risk, vaccination coverage within each site, and differences in the populations who received different vaccine products might confound estimates of vaccine effectiveness. Second, the study period for this analysis occurred before the predominance of the B.1.617.2 (Delta) variant; changes in circulating SARS-CoV-2 variants might affect vaccine effectiveness when assessed over time. Third, persons choosing to receive vaccine later in the rollout might have different risk characteristics than do those vaccinated earlier and might have experienced differences in access to vaccine products by time and location. Finally, this analysis was limited to adults aged ≥65 years, and the results are not generalizable to younger age groups.

This analysis found that all COVID-19 vaccines currently authorized in the United States are highly effective in preventing COVID-19–associated hospitalizations in older adults and also demonstrates the utility of this method in generating a relatively rapid assessment of vaccine performance in the setting of high-quality surveillance and vaccine registry data. Efforts to increase vaccination coverage are critical to reducing the risk for COVID-19–related hospitalization, particularly in older adults.

**Summary**

**What is already known about this topic?**

Clinical trials of COVID-19 vaccines currently authorized for emergency use in the United States (Pfizer-BioNTech, Moderna, and Janssen [Johnson & Johnson]) have shown high efficacy in preventing symptomatic (including moderate to severe) COVID-19.

**What is added by this report?**

Among adults aged 65–74 years, effectiveness of full vaccination for preventing hospitalization was 96% for Pfizer-BioNTech, 96% for Moderna, and 84% for Janssen COVID-19 vaccines; among adults aged ≥75 years, effectiveness of full vaccination for preventing hospitalization was 91% for Pfizer-BioNTech, 96% for Moderna, and 85% for Janssen COVID-19 vaccines.

**What are the implications for public health practice?**

Efforts to increase vaccination coverage are critical to reducing the risk for COVID-19–related hospitalization, particularly in older adults.

## Footnotes

* These authors contributed equally to this report.

† https://www.medrxiv.org/content/10.1101/2021.04.21.21255473v1

§ COVID-NET methodology and sampling scheme: https://www.cdc.gov/coronavirus/2019-ncov/covid-data/covid-net/purpose-methods.html

¶ COVID-NET data included in this analysis were from the following states: California, Colorado, Connecticut, Georgia, Maryland, Michigan, Minnesota, New Mexico, New York, Ohio, Oregon, Tennessee, and Utah.

** IISs are confidential, computerized, population-based systems that collect and consolidate vaccination data from providers in 64 public health jurisdictions nationwide and can be used to track administered vaccines and measure vaccination coverage. https://www.cdc.gov/vaccines/covid-19/reporting/overview/IT-systems.html

††
https://www.cdc.gov/vaccines/covid-19/reporting/vams/program-information.html

§§
https://www.cdc.gov/coronavirus/2019-ncov/vaccines/distributing/about-vaccine-data.html

¶¶ Emergency Use Authorization (EUA) for the Janssen (Johnson & Johnson) vaccine was granted by the Food and Drug Administration on February 26, 2021. EUA was granted for the Pfizer-BioNTech vaccine on December 11, 2020, and for the Moderna vaccine on December 18, 2020.

***
https://www.cdc.gov/nchs/nvss/bridged_race.htm

††† Population vaccine effectiveness is defined as the reduction in disease risk among vaccinated versus unvaccinated persons in the population. Vaccine effectiveness is typically estimated by examining the proportion of persons with disease among those who are vaccinated and the proportion of persons with disease among those who are unvaccinated. If these numbers are difficult to measure or estimate and only case vaccination information is available, then an alternative approach, called the “screening method,” uses estimates of 1) the proportion of persons with disease who are vaccinated and 2) the proportion of persons in the population who are vaccinated. This analysis applied a variation of the screening method through a Poisson regression model, which allows the estimates to account for potential confounding. Specifically, the Poisson regression model uses case counts (both vaccinated and unvaccinated) as the outcome, vaccination status as the exposure variable, and the logarithms of the proportion of vaccinated and unvaccinated persons in the population as offsets. The Poisson model includes the potential confounders time and COVID-NET site as fixed effects because vaccination coverage data are available in each time-by-site stratum. A generalized estimating equation approach with autoregressive correlation structure accommodated daily variations of disease rates and vaccine coverage because this study occurred during a time of very rapid change. Finally, the adjusted vaccine effectiveness estimate was calculated as 1 - exp(β), in which β is the regression coefficient of the vaccination status exposure variable.

§§§ Sampling weights were created based on the probability of selection. Weights were adjusted for nonresponse; adjusted to population catchment totals based on combinations of surveillance site, time period of admission, age, sex, and race/ethnicity via raking procedures; and trimmed to reduce variability.

¶¶¶ 45 C.F.R. part 46.102(l)(2), 21 C.F.R. part 56; 42 U.S.C. Sect. 241(d); 5 U.S.C. Sect. 552a; 44 U.S.C. Sect. 3501 et seq.

****
https://www.medrxiv.org/content/10.1101/2021.04.27.21256193v1

†††† Among COVID-NET patients living in LTCFs, more residents received Pfizer-BioNTech vaccine than received Moderna vaccine, consistent with state distribution through the federal Pharmacy Partnership for Long-Term Care Program. https://www.cdc.gov/vaccines/covid-19/long-term-care/pharmacy-partnerships-faqs.html
